# Omics data integration facilitates target selection for new antiparasitic drugs against TriTryp infections

**DOI:** 10.3389/fphar.2023.1136321

**Published:** 2023-04-06

**Authors:** Martin Rivara-Espasandín, Miranda Clara Palumbo, Ezequiel J. Sosa, Santiago Radío, Adrián G. Turjanski, José Sotelo-Silveira, Dario Fernandez Do Porto, Pablo Smircich

**Affiliations:** ^1^ Departamento de Genómica, Instituto de Investigaciones Biológicas Clemente Estable, Montevideo, Uruguay; ^2^ Departamento de Genética, Facultad de Medicina, Universidad de la República, Montevideo, Uruguay; ^3^ Instituto de Cálculo, Facultad de Ciencias Exactas y Naturales, Universidad de Buenos Aires, Buenos Aires, Argentina; ^4^ Departamento de Química Biológica, Facultad de Ciencias Exactas y Naturales, Universidad de Buenos Aires, Buenos Aires, Argentina; ^5^ Instituto de Química Biológica de la Facultad de Ciencias Exactas y Naturales (IQUIBICEN) CONICET, Ciudad Universitaria, Buenos Aires, Argentina; ^6^ Facultad de Ciencias, Universidad de la República, Montevideo, Uruguay

**Keywords:** trypanosomatids, drug discovery, genomics, neglected disease, target selection

## Abstract

**Introduction:** *Trypanosoma cruzi*, *Trypanosoma brucei*, and *Leishmania spp.*, commonly referred to as TriTryps, are a group of protozoan parasites that cause important human diseases affecting millions of people belonging to the most vulnerable populations worldwide. Current treatments have limited efficiencies and can cause serious side effects, so there is an urgent need to develop new control strategies. Presently, the identification and prioritization of appropriate targets can be aided by integrative genomic and computational approaches.

**Methods:** In this work, we conducted a genome-wide multidimensional data integration strategy to prioritize drug targets. We included genomic, transcriptomic, metabolic, and protein structural data sources, to delineate candidate proteins with relevant features for target selection in drug development.

**Results and Discussion:** Our final ranked list includes proteins shared by TriTryps and covers a range of biological functions including essential proteins for parasite survival or growth, oxidative stress-related enzymes, virulence factors, and proteins that are exclusive to these parasites. Our strategy found previously described candidates, which validates our approach as well as new proteins that can be attractive targets to consider during the initial steps of drug discovery.

## 1 Introduction

Kinetoplastids (Kinetoplastea) are a class of flagellate protists, belonging to the Euglenozoa phylum, characterized by the presence of a structure composed of mitochondrial DNA called kinetoplast. This class consists of many parasites with an extensive host range (Lukeš et al., 2014), divided into 4 orders, one of them being the Trypanosomatida order. Some trypanosomatids constitute an important challenge for human health systems, causing highly prevalent diseases in tropical and subtropical regions, as well as huge economic losses. The main diseases caused by trypanosomatids are Chagas disease (*T. cruzi*) (*Trypanosoma cruzi*), Sleeping sickness (*T. brucei*) (*Trypanosoma brucei*), and Leishmaniasis (*Leishmania* spp.), which are considered neglected diseases, mainly affecting underdeveloped countries⁠ (Hotez et al., 2007).

Having a close phylogenetic relationship (Yazaki et al., 2017), the genome of these three parasites (also known as TriTryps) display high levels of synteny and share a conserved set of genes, most of them arranged in syntenic directional gene clusters (El-Sayed et al., 2005). This fact makes TriTryps share several biological characteristics. Specifically, their life cycle involves replicative and infective stages both in the vector (arthropod) and in the host (mammal) and are extensively described elsewhere (Souza, 2002; Matthews, 2005; Teixeira et al., 2013; Pérez-Molina and Molina, 2018). One of the main differences in their life cycle is the vector species, being triatomine bugs for *T. cruzi*, tse-tse flies for *T. brucei*, and sandflies for *L. major* (*Leishmania major*). Another important difference is the lack of an intracellular replicative stage in the mammal host for *T. brucei* (Matthews, 2005).

Current TriTryps treatments lead to high toxicity and carry significant contraindications, limiting their use. Moreover, used drugs have limited efficiency, and diverse types of drug resistance have been described. Despite this critical situation, drug development projects have been inadequate for reasons ranging from reduced drug discovery efforts by pharmaceutical companies to bad target selection.

Chagas disease is currently treated with chemotherapies based mainly on the use of Nifurtimox and Benznidazole^1^. Both drugs have significant side effects (Rodriques Coura and de Castro, 2002), and are only effective in the early stages of the disease (Nozaki et al., 1996; Wilkinson et al., 2008).

Four drugs are currently used during Sleeping sickness treatment: pentamidine, suramin, melarsoprol, and eflornithine^2^. All of them have shown dose-dependent side effects, with varying toxicity levels (Babokhov et al., 2013). The oral drug Fexinidazole was recently approved to combat both the early and late stages of the disease (Mesu et al., 2018).

Regarding leishmaniasis, amphotericin B, miltefosine, and pentamidine, are the most used drugs^3^ and have significant side effects (Oliveira et al., 2011). Its most severe form, visceral leishmaniasis, is treated by pentavalent antimonials (Aronson et al., 2017), which can be cardiotoxic and cause arrhythmias (Sundar and Chakravarty, 2010).

All these issues regarding current treatments highlight the importance of investing time and resources in the research and development of new drugs and treatments.

The search and development of new drugs is a long and expensive process (DiMasi et al., 2016). Reasons behind unsuccessful new antimicrobial development projects range from inadequate selection of the molecular targets to a lack of innovation. In this context, increasingly available omics data for multiple pathogens has created new drug discovery and development opportunities to fight infectious diseases (Serral et al., 2021; Schottlender et al., 2022).

A widely used approach in computational screening is target-based drug discovery, a strategy focused on seeking compounds that can fit a specific target, and are not based on general characteristics (Croston, 2017). In this way, compounds that can bind and modify the activity of a specific target are sought, saving time and resources.

Here, we report on the application of a multidimensional data integration strategy to prioritize drug targets in TriTryps. By combining different layers of omic-scale information, which include genomic, transcriptomic, metabolic, and protein structural data sources, we were able to delineate candidate proteins with relevant features for target selection in the development of new drugs capable of combating different groups of clinical relevance within the order of trypanosomatids. We expect our results to be particularly useful in accelerating the initial steps of drug discovery through the identification of attractive targets.

## 2 Materials and methods

All the performed analyses are based either on the *in silico* calculation of selected features for each protein within the trypanosomatid proteome or on the integration of available data. Below, we briefly describe the applied protocols to compute the desired properties. Further details on the data sources and methods can be found in the corresponding references.

### 2.1 Trypanosomatids genomes

All the analyses were performed using genomes available on TriTrypDB (release 46). *Trypanosoma cruzi*: CL Brener Esmeraldo-like, *T. brucei*: DAL972, and *L. major*: Friedlin.

### 2.2 Structural assessment of druggability

Experimental structures for TriTryps were obtained from the Protein Data Bank (PDB). For all the remaining proteins, we attempted to predict their structure by homology modeling as described in (Sosa et al., 2018). Structural models, template information and validation of the models are available on Target Pathogen^4^ database.

The structural druggability of *T. cruzi*, *T. brucei* and *L. major* proteins was assessed using the fpocket program (Schmidtke et al., 2010) and Druggability Score (*DS*) index (Schmidtke and Barril, 2010; Schmidtke et al., 2010) as defined in (Sosa et al., 2018). This assessment was performed both on crystals and on homology-based models.

### 2.3 Evaluation of potential off-target effects

All the proteins of TriTryps were blasted against the human proteome (NCBI assembly access GCF_000001405.36) to identify close human homologous. Hits with an identity greater than 40% and E-value less than 10^–5^ were ruled out, as they may share a high degree of structural preservation that could produce side effects if the parasite protein is used as target of a putative drug.

### 2.4 Metabolic network analysis

The PathoLogic module within Pathway Tools v. 20.0 (Karp et al., 2002) environment was used to build each metabolic network using the respective annotated genome as input. The metabolic reconstruction included the determination of reaction-protein-gene associations, which are primarily based on the corresponding enzyme commission (EC) number. EC number annotations were previously added to the genomic annotation. After manual curation of the metabolic networks, choke-point (CP) analysis was conducted within Pathway Tools. Choke-point reactions (CPs) are those that either uniquely produce or consume a given product or substrate, respectively (Yeh et al., 2004). In this sense, it is assumed that CP blockade may lead to the lack of an essential compound or the accumulation of a toxic metabolite in the cell; thus, these types of reactions have great significance in drug targeting.

### 2.5 Target prioritization pipeline

All previously calculated data was integrated in Target Pathogen. Target Pathogen is our own web server developed for drug target prioritization. This webserver was previously used to obtain attractive targets for drug development projects in other relevant pathogens (Defelipe et al., 2016; Ramos et al., 2018; Farfán-López et al., 2020; Palumbo et al., 2022; Serral et al., 2022). The integrated data was used to obtain a list of drug target candidates. In this sense, proteins of the studied TriTryps were filtered and ranked using the Target-Pathogen database. At first, proteins with *DS* < 0.5 (non-druggable or poorly druggable proteins) and proteins with cross-reaction potential with the human host were filtered out. We then defined a scoring function as follows to assign a score to each protein. This equation assigns a score that defines a protein’s potential as a drug target based on its druggability, human off-target, and metabolic context (CPs).

Scores are assigned using the following function:

S=1∗DS+1∗HOT+5∗CP

where *DS* is the Druggability Score, *HOT* is the Human Off-Target Score and *CP* defines whether the protein is associated with a CP reaction. *HOT* reflects the results of a blastp search of the pathogen protein in the human proteome database with the scale: 1-identity of the best hit of the queried protein and the human proteome. *DS* and *HOT* can take values between 0 and 1, while *CP* is equal to 1 if the protein is involved in a CP reaction, otherwise is set to zero. We have given the *CP* parameter a weight of 5, so that the proteins involved in CPs (usually essential proteins for the organism metabolism) lead the ranking.

### 2.6 Protein clustering

For clustering proteins among these 3 parasites, we used the MMSeqs2 software (Steinegger and Söding, 2017). A minimum coverage of 70%, a sequence identity greater than 50% and an E-value less than 0.001 were used as cut-off points to determine a significant hit. Alignment method number 3 was used, as it is described as the most accurate approach, and clustering mode 0 which uses the Greedy Set Cover algorithm was selected.

### 2.7 Cluster polishing

Once the clusters were obtained, it was observed that many of them contained more than one paralog gene per parasite. Common clusters were reduced to a single gene from each of the parasites, looking for candidates with similar *DS* and *HOT*. To carry out this cluster reduction, genes with *DS* < 0.5 and *HOT* < 0.7 were first removed. Then, *DS* standard deviation (*stdDS*) and *HOT* standard deviation (*stdHOT*) were calculated for all combinations involving a single gene from each of the parasites. Finally, the combinations with *stdDS* > 0.1 and *stdHOT* > 0.1 were filtered out, obtaining triplets with similar *DS* and *HOT* in each of the clusters. As result, each cluster is composed of similar proteins from each of the parasites with *DS* ≥ 0.5 and *HOT* ≥ 0.7. Finally, if a cluster has two or more combinations that pass these filters, the one with the highest average *DS* was kept.

### 2.8 Cluster prioritization pipeline

The defined clusters were ranked based on the *S-score* obtained for each gene in each of the parasites, that was calculated as described in the “Target prioritization pipeline” section. Using the *S-score* of each of the three genes belonging to a cluster, the mean and the standard deviation were calculated. With these values, a ranking of clusters was determined using the mean in descending order as first factor and the standard deviation in ascending order as a second factor.

### 2.9 Gene expression analysis

Drugs affect the parasites during stages in the mammalian host, therefore it is important to analyze candidates’ expression at these life cycle stages. For *T. cruzi*, they are the trypomastigote and amastigote stages, for *T. brucei* the blood trypomastigote stage, and for *L. major* the metacyclic promastigote and amastigote stages. The gene expression analysis was performed using data from Li et al. (Li et al., 2016) for *T. cruzi*, data from Naguleswaran et al. (Naguleswaran et al., 2018) for *T. brucei* and data from Inbar et al. (Inbar et al., 2017) for *L.* major. Candidate mRNA levels were evaluated at the trypomastigote and amastigote (48 h post-infection) stages for *T. cruzi*, at the slender and stumpy forms of blood trypomastigote stage for *T. brucei* and at the metacyclic promastigote and amastigote stages for *L. major*. We compared the expression of our candidates with the total mRNAs for each stage of the life cycle.

For *L. major* raw reads were downloaded, trimmed and Kallisto version 0.46.1 (Bray et al., 2016) was used to obtain expression estimates for the reference transcriptome (TriTrypDB v.6.1 *L. major* Friedlin) (Aslett et al., 2010). Read pseudo counts were normalized by sequencing depth for each replicate. For *T. cruzi* and *T. brucei* analysis counts tables were available and used in this work. Log2 transformation and quantile normalization were applied to all counts tables. We considered a gene to be expressed if this value is greater than 0.

### 2.10 Gene ontology terms enrichment analysis

Once the candidate list was obtained, functional analyses were performed to better understand the roles of these proteins in the parasite’s biology. We first evaluated the Gene Ontology (GO) terms overrepresentation, using the trypanosomatid database TritrypDB (Aslett et al., 2010). The analysis was performed for Molecular Function, Biological Process and Cellular Component ontology terms with default parameters, reporting both curated and computed terms, and using an FDR cut-off of 0.05 to determine enrichment.

### 2.11 Text mining

To better understand the biological potential of the candidates as putative drug targets, a literature search was performed, aided by IdMiner^5^ software. It allows linking candidate identifiers to published articles and search for overrepresented terms. The search term “drug” was used to find an association between our candidates and drug research and development articles.

## 3 Results and discussion

### 3.1 Omic data integration allows the prioritization of putative drug targets in TriTryps

**FIGURE 1:**
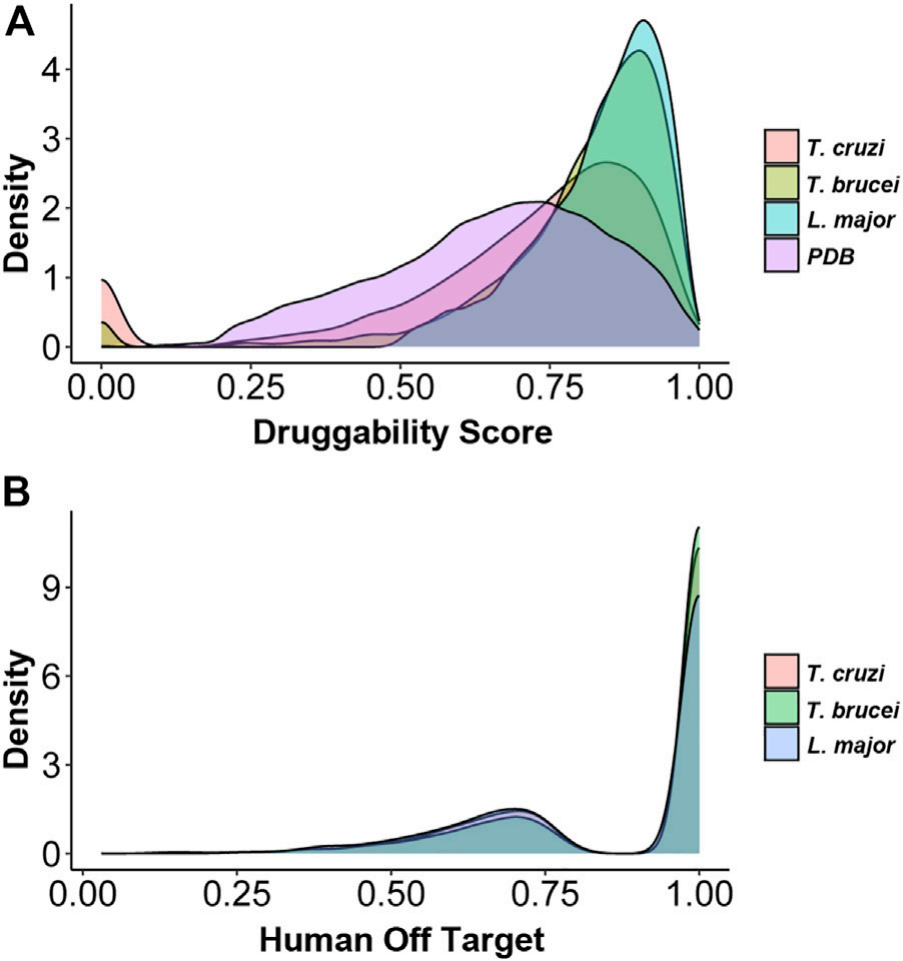
Distribution of protein Druggability Scores (*DSs*) and Human Off-Target Scores (*HOT*) in each of the parasites **(A)** Density plots showing the distribution of *DSs* ​​for *Trypanosoma cruzi*, *Trypanosoma brucei*, *Leishmania major* proteins as well as the complete PDB database for comparison **(B)** Density plots showing the distribution of *HOT* for *Trypanosoma cruzi*, *Trypanosoma brucei* and *Leishmania major* proteins.

*DS* of each protein pocket were calculated for all protein structures we were able to obtain for *T. cruzi*, *T. brucei*, and *L. major*. *DS* was then assigned to 4592, 4197, and 6656 proteins from *T. cruzi*, *T. brucei* and *L. major* respectively **(** Table 1). The difference in the number of proteins assigned a *DS* for *L. major* is striking and possibly reflects that both trypanosomes are more phylogenetically related, but this observation will require further analysis. Figure 1A shows the distribution of *DS* for the three parasites as well as for the RCSB Protein Data Bank (PDB), with a clear enrichment in highly druggable proteins for all the TriTryps.

**TABLE 1:** Classification of *Trypanosoma cruzi*, *Trypanosoma brucei* and *Leishmania major* structures. Proteins were grouped based on their Druggability Score (*DS*) into four sets: non-druggable (0.0 ≤ *DS* < 0.2), poorly druggable (0.2 ≤ *DS* < 0.5), druggable (0.5 ≤ *DS* < 0.7) and highly druggable (0.7 ≤ *DS* ≤ 1.0). Additionally, we classified all structures experimentally obtained in complex with a drug-like compound or an inhibitor (ED+) or without a binding drug (ED-), and all models whose template was co-crystallized with a drug (MD+) or not (MD-). Total values are shown in bold.

	*L. major*					*T. cruzi*					*T. brucei*				
	ED+	ED-	MD+	MD-	Total	ED+	ED-	MD+	MD-	Total	ED+	ED-	MD+	MD-	Total
Non-druggable	1	3	32	153	**189**	0	19	20	300	**339**	0	3	2	68	**73**
Poorly druggable	7	11	54	133	**205**	0	3	57	287	**347**	0	4	8	104	**116**
Druggable	23	11	270	443	**747**	5	6	232	790	**1033**	5	12	63	446	**526**
Highly druggable	35	30	2833	2617	**5515**	20	19	638	2196	**2873**	45	44	770	2623	**3482**
Total	**66**	**55**	**3189**	**3346**	**6656**	**25**	**47**	**947**	**3573**	**4592**	**50**	**63**	**843**	**3241**	**4197**

Given that trypanosomatids are ancient eukaryotes, the observed similarity of their proteins to their human counterparts is low (Figure 1B) (Burki et al., 2020). However, this analysis allowed us to exclude highly conserved proteins that are not suitable candidates for drug targeting candidates as they are more likely to exhibit off-target effects.

Metabolic network reconstruction was performed with Pathway Tools followed by manual curation. TriTryps networks were analyzed allowing the determination of CPs. For *T. cruzi* 575 proteins were assigned to reactions distributed across 98 metabolic pathways. A total of 466 proteins participated in CPs, of which 70 were associated with producing CPs, 374 with strictly consuming ones and 22 were mapped with both types. *Trypanosoma brucei* metabolic network is composed of 234 predicted pathways. From a total of 386 proteins involved in reactions, 156 were annotated as CPs. 57 were classified as production CPs, 58 as consuming ones and 41 on both producing and consuming sides.

Finally, *L. major* metabolic model resulted in 142 pathways, composed of 307 proteins assigned to reactions, with 135 classified as CPs. A total of 45 and 62 were annotated as producing and consuming CPs, respectively. Additionally, 28 proteins were classified as CPs on both types.

**FIGURE 2:**
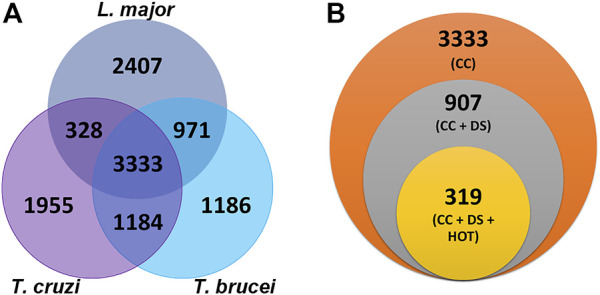
Selection of common drug target candidates in TriTryps **(A)** Clusters obtained by using MMseqs2. Venn diagram representing the intersection of similar genes for the three parasites **(B)** Potential drug targets common to *Trypanosoma cruzi*, *Trypanosoma brucei* and *Leishmania major*. Each circle depicts the number of proteins obtained after applying the indicated filter. Orange: 3,333 common clusters for the three parasites. Gray: 907 common clusters where the three proteins have a *DS* ≥ 0.5. Yellow: 319 common clusters where the three proteins also have a *HOT* ≥ 0.7.

The shared biological characteristics of TriTryps give us the opportunity to search for common druggable proteins, enabling the design of drugs that might be effective against more than one species. Similarity clustering analysis resulted in 3,333 protein groups common to *T. cruzi*, *T. brucei* and *L. major* (Figure 2A).

By using the tools included in Target Pathogen database, we combined all the previous results and filtered the candidates with *DS* ≥ 0.5 and *HOT* ≥ 0.7, obtaining a list of 319 protein clusters with interesting features such as druggable pockets and low sequence similarity with human proteins (Figure 2B). Of these, 82 are annotated as hypothetical proteins in all three TriTryps and 139 have this annotation in at least one of them. The rest of the clusters are functionally annotated for the three parasites.

**FIGURE 3:**
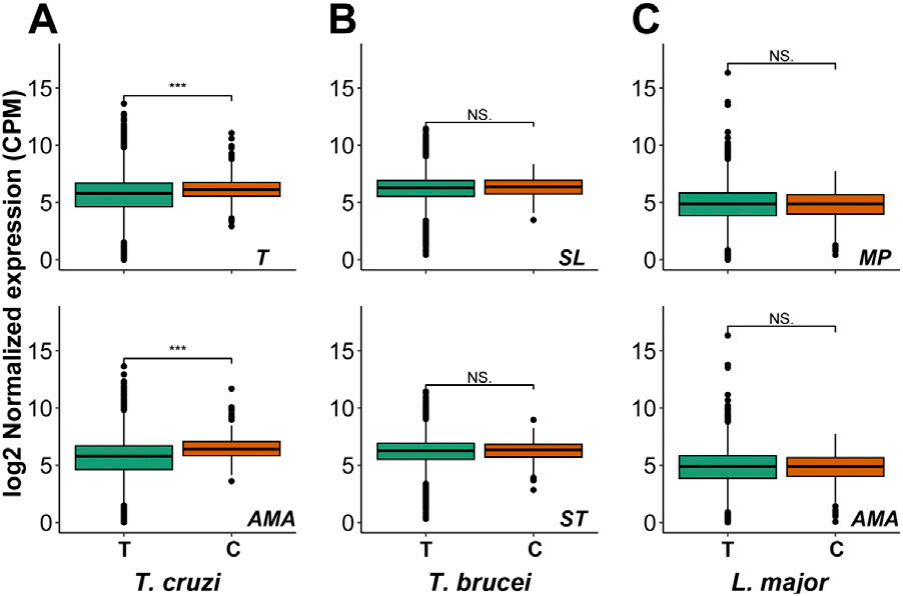
Candidate genes expression in life cycle stages. Box plots showing the mRNA levels of the total genes (T) and of our drug target candidates (C) in **(A)** *T. Cruzi* trypomastigotes (T) and amastigotes (AMA) **(B)** *Trypanosoma brucei* slender (SL) and stumpy (ST) forms of blood trypomastigotes and **(C)** *Leishmania major* metacyclic promastigotes (MP) and amastigotes (AMA). Mann–Whitney–Wilcoxon test was performed to compare the different gene sets [*p*-value <0.001 (***); *p*-value <0.05 (*); no significance (NS.)]. Only expressed genes are plotted (see Materials and Methods).

Since drugs should be effective against the parasite forms present in the mammalian host, we analyzed the gene expression patterns of the candidate proteins at these stages. We observed that the mRNAs coding for these proteins are expressed in all analyzed forms for the three parasites (Figure 3), excepting LmjF.01.0140 in both analyzed *L. major* stages, LmjF.34.0190 in *L. major* amastigote stage and Tbg.972.2.3880, Tbg972.8.3790 for both *T. brucei* stages. These expression values in the mammalian stages make them interesting drug target candidates.

### 3.2 Protein candidates are involved in a wide variety of relevant biological processes

The GO analysis does not report any enrichment either for Molecular Function or for Biological Process terms. For the Cellular Components ontology, only mitochondrion (GO:0005739, FDR <0.05) and kinetoplast (GO:0020023, FDR <0.05) were reported as enriched in our list. This is an interesting observation as proteins involved in the mitochondrial oxidative stress response are considered putative drug targets (see discussion below). Proteins related to the kinetoplast are also reasonable candidates given the uniqueness and relevance of this structure in the biology of the TriTryps. Manual inspection of the list revealed two proteins with relevant features that are discussed in detail below (TcCLB.510,295.30, Tbg972.10.6190, LmjF.36.0700); (TcCLB.506,287.200, Tbg972.7.500, LmjF.26.1340).

No functional relationships are expected to be *a priori* in the list, so, as expected, no other evident functional relationship is observed among the candidates. This prompted us to manually inspect the functions of interesting candidates on our list and discuss their relevance as potential drug targets (Supplementary Table S1 **)**. As can be observed, their functions are varied but they are involved in key biological aspects of parasite biology.

#### 3.2.1 Essential proteins for growth or survival

Some of the ranked proteins or the pathways in which they are involved have already been described as essential for the growth or survival of the parasite. This is the case of Pseudouridine tRNA synthases (TcCLB.507,639.50, Tbg972.10.7460, LmjF.36.1660), enzymes that carry out post-transcriptional modifications in tRNAs. They have not been extensively studied in trypanosomatids, except for CBF5 in *T. brucei* (Barth et al., 2005; Chikne et al., 2016; Rajan et al., 2019). However, Michaeli et al. (Barth et al., 2005) demonstrated that parasite growth is inhibited by silencing the expression of CBF5, evidencing the essentiality of this protein.

Farnesyl Transferases (PFT) (TcCLB.507,257.70, Tbg972.3.4930, LmjF.29.1950) are enzymes involved in protein prenylation, a post-translational modification relevant to bind proteins to cell membranes (Zhang and Casey, 1996). Many PFT inhibitors have been tested against trypanosomatids, showing a significant reduction in growth rate (Yokoyama et al., 1998; Eastman et al., 2006). Several PFT inhibitors have been developed as anticancer compounds (Wang et al., 2017), so a “piggy-back” approach has been used as a starting point to develop new antiparasitic drugs (Ohkanda et al., 2004; Eastman et al., 2006).

S-adenosylmethionine decarboxylase (AdoMetDC) is an important enzyme involved in the polyamines (spermidine) synthesis pathway (Bacchi, 1981). In trypanosomatids AdoMetDC is catalytically activated by the formation of a heterodimer with a proenzyme (TcCLB.509167.110, Tbg972.6.4250, LmjF.30.3120) that stimulates its activity (Willert et al., 2007; Willert and Phillips, 2009). This proenzyme is a catalytically dead paralog, required for an effective AdoMetDC´s activity and apparently unique to the trypanosomatid group (Willert et al., 2007; Willert and Phillips, 2008). Both AdoMetDC and the proenzyme are essential for the growth and survival of *T. brucei* parasites (Willert and Phillips, 2008), and inhibitors of the reaction has been developed and tested (Bitonti et al., 1990; Bacchi et al., 1992; Barker et al., 2009) making this proenzyme an interesting drug target.

DNA ligase Kα (TcCLB.506,287.200, Tbg972.7.500, LmjF.26.1340) is a protein involved in the kinetoplast DNA (kDNA) replication (Jensen and Englund, 2012). It has been shown to be essential for the normal growth of procyclic *T. brucei* parasites and its knockdown by RNAi leads to rapid loss of the kDNA from the cell (Downey et al., 2005). It is important to mention that some trypanosomatid lineages have lost their kDNA, giving rise to natural dyskinetoplastic parasites (Schnaufer et al., 2002), This is possible as bloodstream forms do not depend on the oxidative phosphorylation, however we do not have experimental data to know the effect of knocking this enzyme in *T. cruzi* or Leishmania where kDNA loss might be lethal or might affect cell growth (Girard et al., 2016; Yang et al., 2016). Moreover, *T brucei* stumpy bloodstreams lifespan is reduced when lacking kDNA (Dewar et al., 2018). Given that kDNA is exclusive to kinetoplastids, kinetoplast-related processes have already been discussed as potential drug targets (Motta, 2008).

#### 3.2.2 Oxidative stress response

Enzymes that protect the parasite from host-generated oxidative stress are key players during trypanosomatid infections.

Superoxide dismutases (SOD) (TcCLB.511,735.60, Tbg972.6.3810, LmjF.30.2770) are antioxidant metalloenzymes that dismutate O_2_^−^ into molecular oxygen and hydrogen peroxide (H_2_O_2_), thereby scavenging superoxide radicals (McCord and Fridovich, 1969). Trypanosomatids have four Fe-SOD isoenzymes (Dufernez et al., 2006), a type that has been found only in prokaryotes, protozoans and chloroplasts. Antisense-mediated knockdown of Fe-SOD in Leishmania parasites showed increased susceptibility to oxidative stress agents and reduced growth and survival within macrophages (Ghosh et al., 2003). Fe-SOD are also involved in the virulence promotion in Leishmania and *T. cruzi* (Mittra et al., 2017; Martínez et al., 2019). Fe-SOD is also overexpressed in *T. cruzi* parasites resistant to Benznidazole (Nogueira et al., 2006). Moreover, inhibitors of this enzyme have been developed, showing a decrease in the infection rate and cell growth for *T. cruzi* (Sanz et al., 2008).

Glutathione S-transferases or glutaredoxins (TcCLB.508,265.10, Tbg972.7.3850, LmjF.14.1480) are oxidoreductases that belong to the thioredoxin family (Qi and Grishin, 2004). They are important for TriTryps redox homeostasis and iron metabolism (Comini et al., 2008; Marquez et al., 2010). Some of them have been shown to be essential for parasite growth (Ceylan et al., 2010; Ebersoll et al., 2018).

The dihydrolipoamide dehydrogenase enzyme (LipDH) (TcCLB.509,379.10, Tbg972.4.5230, LmjF.31.2650) catalyzes the reversible conversion of dihydrolipoamide and NAD^+^ into lipoamide and NADH and is part of three mitochondrial matrix complexes involved in the citric acid cycle (Lohrer and Krauth-Siegel, 1990; Portela and Stopopani, 1991; Schöneck et al., 1997). Given this reversible reaction, LipDH can generate free thiols (dihydrolipoate) that act as free radical quenchers (Kagan et al., 1992). Its essentiality has been shown in both stages of the life cycle in *T. brucei* (Roldán et al., 2011). In addition, LipDH protein has an increased activity and is overexpressed at the mRNA and protein level, when comparing Benznidazole resistant *T. cruzi* populations with susceptible populations (dos Santos et al., 2016). This and other properties make LipDH an interesting drug target candidate (Krauth-Siegel and Schöneck, 1995).

#### 3.2.3 Virulence factors

Virulence and infectivity are key qualities when evaluating the relevance of different parasite strains.

Acid phosphatases (TcCLB.511,589.74, Tbg972.11.13180, LmjF.36.6480) are related to variations in virulence levels in *L. major* (Baghaei and Mesripour, 2015). In *T. brucei*, the membrane acid phosphatase MBAP-1 is reported to be essential for the parasite’s survival (Engstler et al., 2005), while in *Leishmania mexicana* this enzyme is not essential at stages in the host (Benzel et al., 2000).

The ABCG1 membrane transporter (TcCLB.506,579.10, Tbg972.10.9440, LmjF.27.1700) belongs to the ABC transporters family, which are proteins involved in translocation of solutes across cell membranes and are present in all phyla (Jones and George, 2004). Using a double knock-out approach, ABCG1 has been shown to be a relevant protein in the infectivity and virulence of *L. major* (Manzano et al., 2017). In *T. cruzi* this protein is involved in the resistance to Benznidazole (Zingales et al., 2015).

#### 3.2.4 Parasite specific proteins

When looking for potential drug targets, the absence of a similar protein in humans is a key quality, as off-target effects are potentially reduced.

RNA editing is a post-transcriptional RNA processing mechanism, unique to trypanosomatids that involves the insertion and removal of uridine nucleotides directed by a guide RNA (Madison-Antenucci et al., 2002; Stuart et al., 2005). Most mitochondrial mRNAs require editing, making it an essential process in TriTryps (Madison-Antenucci et al., 2002). Many components of the editing complex are essential for parasite survival and different inhibitors have been tested against them [reviewed in (Salavati et al., 2011)]. Our candidate MP18 (TcCLB.510,295.30, Tbg972.10.6190, LmjF.36.0700) is one of the proteins that make up the editing complex and is essential for *T. brucei* survival (Law et al., 2007; Tarun et al., 2008), making it a really interesting target.

Asparagine synthetases (AS) (TcCLB.503,899.90, Tbg972.7.1080, LmjF.26.0830) are responsible for the synthesis of Asparagine (Asn) from aspartate. There are two main types of AS, type B present in prokaryotes and eukaryotes, and type A present in prokaryotes and strikingly also in kinetoplastids and other protozoa (Faria et al., 2016). Our candidate protein is type A, which makes it very interesting given its absence in humans (Manhas et al., 2014). Regarding its essentiality, in *T. brucei* it was found that in presence of Asn parasites with an AS knockdown are fully functional and their survival, infectivity and virulence were not affected (Loureiro et al., 2013). The same was shown for *Leishmania infantum* in null mutants obtained by gene replacement (Faria et al., 2016). On the other hand, in *Leishmania donovani*, it was observed through gene replacement studies that this gene is essential for parasite viability (Manhas et al., 2014). These studies show that the essentiality of this potential target in trypanosomatids is unclear. Cordeiro da Silva et al. (Loureiro et al., 2013) suggest that the only possible combination would be a therapy that should involve an inhibitor of this enzyme as well as a depletion of extracellular Asn (e.g., L-asparaginase) or a blockade of Asn transporters. According to these researchers, this combination is very difficult, since it requires a lot of development and logistics resources, and the use of two different drugs can lead to the development of resistance. They also add that treatment with L-asparaginase has been seen to cause significant adverse effects in cancer, (Appel et al., 2008; Cohen et al., 2010).

Oligopeptidase B (OpB) (TcCLB.511557.10, Tbg972.11.14320, LmjF.06.0340) is a serine protease, present in prokaryotes (Polgár, 1997; Fenno et al., 2001; Morty et al., 2002; Yan et al., 2006), trypanosomatids (Morty et al., 2001; Morty et al. 2005; de Matos Guedes et al. 2007; Swenerton et al., 2011), some plants (Matheson et al., 1995; Bagarozzi et al., 1998; Guo et al., 1998) and is predicted in Fungi genomes. So far, they have not been described in mammals. In *T. cruzi* it is involved in the invasion of non-phagocytic cells *via* lysosomes (Burleigh et al., 1997), one of the different pathways that the parasite uses to infect cells (Caradonna and Burleigh, 2011; Barrias et al., 2013; Ferri and Edreira, 2021). This pathway involves a release of intracellular Ca^2+^ in the host cells, which determines an increase in the presence of lysosomes in the membrane, where *T. cruzi* enters to differentiate later to the amastigote stage. OpB generates an agonist that binds to a membrane receptor that determines Ca^2+^ signalling and subsequent invasion. In this study, it has been seen that treatment with anti-OpB antibodies significantly reduces the invasive capacity of *T. cruzi*, something that was later confirmed by *in vivo* experiments in mice.

In conclusion, we have evidence that many of the candidates are reasonable drug targets or have already been proposed as such, validating our bioinformatic approach. Interestingly our work provides an opportunity to explore many new proteins that, to our knowledge, have not yet been considered as potential targets.

## 4 Conclusion

Neglected diseases have been poorly studied for many years, the main consequence being ineffective treatments with serious side effects. Aiming to reverse this situation, an exhaustive search for potential drug candidates was recently initiated, within the framework of the non-profit organization DNDi^6^.

In this work we seek to have a first visualization of proteins common to *T. cruzi*, *T. brucei*, and *L. major*, which have interesting properties to be potential drug targets, in order to define certain candidates on which to focus future studies. For this, we used sequence clustering methodologies to search for shared orthologs among the three parasites, and we evaluated their druggability, human off-targeting, expression level, as well as their importance for the parasite’s biology, using the Target Pathogen and bibliographic searches. The method depends on available data, so the analysis could be improved in new versions when more data becomes accessible. Thus, we present a ranked list of 319 common candidates for TriTryps, with interesting features as potential drug targets. Further studies would be needed to experimentally validate these candidates. We expect that this work will pave the process of drug discovery against TriTryps.

## Data Availability

The datasets presented in this study can be found in online repositories. The names of the repository/repositories and accession number(s) can be found below: http://target.sbg.qb.fcen.uba.ar/patho/.

## Footnotes

1 WHO (2022a). Chagas disease. Available at: https://www.who.int/news-room/fact-sheets/detail/chagas-disease-(american-trypanosomiasis) [Accessed 6 July 2022]

2 WHO (2022b). Trypanosomiasis, human African (sleeping sickness). Available at: https://www.who.int/news-room/fact-sheets/detail/trypanosomiasis-human-african-(sleeping-sickness) [Accessed 6 July 2022]

3 WHO Expert Committee on the Control of the Leishmaniases and World Health Organization (2010). Control of the leishmaniases: report of a meeting of the WHO Expert Commitee on the Control of Leishmaniases, Geneva, 22–26 March 2010. Control Las Leishmaniasis Inf. Una Reunión Com. Expert. OMS Sobre El Control Las Leishmaniasis Ginebra 22 26 Marzo 2010. Available at: https://apps.who.int/iris/handle/10665/44412 [Accessed 6 July 2022]

4 Target Pathogen. Available at: http://target.sbg.qb.fcen.uba.ar/patho/[Accessed 30 December 2022]

5 Radio, S (2022). Sradiouy/IdMiner. Available at: https://github.com/sradiouy/IdMiner [Accessed 30 December 2022]

6 DNDi—Best science for the most neglected (2019). Available at: https://dndi.org/[Accessed 30 December 2022]

## References

[B1] Appel I. M. Hop W. C. J. van Kessel-Bakvis C. Stigter R. Pieters R. (2008). L-Asparaginase and the effect of age on coagulation and fibrinolysis in childhood acute lymphoblastic leukemia. Thromb. Haemost. 100, 330–337. 10.1160/TH07-10-0620 18690355

[B2] Aronson N. Herwaldt B. L. Libman M. Pearson R. Lopez-Velez R. Weina P. (2017). Diagnosis and treatment of leishmaniasis: Clinical practice guidelines by the infectious diseases society of America (IDSA) and the American society of tropical medicine and Hygiene (ASTMH). Am. J. Trop. Med. Hyg. 96, 24–45. 10.4269/ajtmh.16-84256 27927991 PMC5239701

[B3] Aslett M. Aurrecoechea C. Berriman M. Brestelli J. Brunk B. P. Carrington M. (2010). TriTrypDB: A functional genomic resource for the trypanosomatidae. Nucleic Acids Res. 38, D457–D462. 10.1093/nar/gkp851 19843604 PMC2808979

[B4] Babokhov P. Sanyaolu A. O. Oyibo W. A. Fagbenro-Beyioku A. F. Iriemenam N. C. (2013). A current analysis of chemotherapy strategies for the treatment of human African trypanosomiasis. Pathog. Glob. Health 107, 242–252. 10.1179/2047773213Y.0000000105 23916333 PMC4001453

[B5] Bacchi C. J. (1981). Content, synthesis, and function of polyamines in trypanosomatids: Relationship to chemotherapy. J. Protozool. 28, 20–27. 10.1111/j.1550-7408.1981.tb02798.x 6788943

[B6] Bacchi C. J. Nathan H. C. Yarlett N. Goldberg B. McCann P. P. Bitonti A. J. (1992). Cure of murine *Trypanosoma brucei rhodesiense* infections with an S-adenosylmethionine decarboxylase inhibitor. Antimicrob. Agents Chemother. 36, 2736–2740. 10.1128/AAC.36.12.2736 1482141 PMC245537

[B7] Bagarozzi D. A. Potempa J. Travis J. (1998). Purification and characterization of an arginine-specific peptidase from ragweed (ambrosia artemisiifolia) pollen. Am. J. Respir. Cell Mol. Biol. 18, 363–369. 10.1165/ajrcmb.18.3.2825 9490654

[B8] Baghaei M. Mesripour M. (2015). Characterization of acid phosphatase in the promastigotes of three isolates of *Leishmania major* . Iran. J. Med. Sci. 28, 1–8.

[B9] Barker R. H. Liu H. Hirth B. Celatka C. A. Fitzpatrick R. Xiang Y. (2009). Novel S-adenosylmethionine decarboxylase inhibitors for the treatment of human african trypanosomiasis. Antimicrob. Agents Chemother. 53, 2052–2058. 10.1128/AAC.01674-08 19289530 PMC2681509

[B10] Barrias E. Carvalho T. De Souza W. (2013). Trypanosoma cruzi: Entry into mammalian host cells and parasitophorous vacuole formation. Front. Immunol. 4, 186. 10.3389/fimmu.2013.00186 23914186 PMC3730053

[B11] Barth S. Hury A. Liang X. Michaeli S. (2005). Elucidating the role of H/ACA-like RNAs in trans-splicing and rRNA processing via RNA interference silencing of the *Trypanosoma brucei* CBF5 pseudouridine synthase. J. Biol. Chem. 280, 34558–34568. 10.1074/jbc.M503465200 16107339

[B12] Benzel I. Weise F. Wiese M. (2000). Deletion of the gene for the membrane-bound acid phosphatase of *Leishmania mexicana* . Mol. Biochem. Parasitol. 111, 77–86. 10.1016/S0166-6851(00)00306-6 11087918

[B13] Bitonti A. J. Byers T. L. Bush T. L. Casara P. J. Bacchi C. J. Clarkson A. B. (1990). Cure of *Trypanosoma brucei brucei* and *Trypanosoma brucei rhodesiense* infections in mice with an irreversible inhibitor of S-adenosylmethionine decarboxylase. Antimicrob. Agents Chemother. 34, 1485–1490. 10.1128/AAC.34.8.1485 1977366 PMC171857

[B14] Bray N. L. Pimentel H. Melsted P. Pachter L. (2016). Near-optimal probabilistic RNA-seq quantification. Nat. Biotechnol. 34, 525–527. 10.1038/nbt.3519 27043002

[B15] Burki F. Roger A. J. Brown M. W. Simpson A. G. B. (2020). The new tree of eukaryotes. Trends Ecol. Evol. 35, 43–55. 10.1016/j.tree.2019.08.008 31606140

[B16] Burleigh B. A. Caler E. V. Webster P. Andrews N. W. (1997). A cytosolic serine endopeptidase from *Trypanosoma cruzi* is required for the generation of Ca2+ signaling in mammalian cells. J. Cell Biol. 136, 609–620. 10.1083/jcb.136.3.609 9024691 PMC2134300

[B17] Caradonna K. L. Burleigh B. A. (2011). Mechanisms of host cell invasion by Trypanosoma cruzi. Adv. Parasitol. 76, 33–61. 10.1016/B978-0-12-385895-5.00002-5 21884886

[B18] Ceylan S. Seidel V. Ziebart N. Berndt C. Dirdjaja N. Krauth-Siegel R. L. (2010). The dithiol glutaredoxins of african trypanosomes have distinct roles and are closely linked to the unique trypanothione metabolism. J. Biol. Chem. 285, 35224–35237. 10.1074/jbc.M110.165860 20826822 PMC2966136

[B19] Chikne V. Doniger T. Rajan K. S. Bartok O. Eliaz D. Cohen-Chalamish S. (2016). A pseudouridylation switch in rRNA is implicated in ribosome function during the life cycle of *Trypanosoma brucei* . Sci. Rep. 6, 25296. 10.1038/srep25296 27142987 PMC4855143

[B20] Cohen H. Bielorai B. Harats D. Toren A. Pinhas-Hamiel O. (2010). Conservative treatment of L -asparaginase-associated lipid abnormalities in children with acute lymphoblastic leukemia: Asparaginase Induced Hypertriglyceridemia. Pediatr. Blood Cancer 54, 703–706. 10.1002/pbc.22305 20063421

[B21] Comini M. A. Rettig J. Dirdjaja N. Hanschmann E.-M. Berndt C. Krauth-Siegel R. L. (2008). Monothiol glutaredoxin-1 is an essential iron-sulfur protein in the mitochondrion of african trypanosomes. J. Biol. Chem. 283, 27785–27798. 10.1074/jbc.M802010200 18669638

[B22] Croston G. E. (2017). The utility of target-based discovery. Expert Opin. Drug Discov. 12, 427–429. 10.1080/17460441.2017.1308351 28306350

[B23] de Matos Guedes H. L. Carneiro M. P. D. de Oliveira Gomes D. C. Rossi-Bergmann B. De-Simone S. G. (2007). Oligopeptidase B from *Leishmania amazonensis*: Molecular cloning, gene expression analysis and molecular model. Parasitol. Res. 101, 865–875. 10.1007/s00436-007-0630-8 18074461

[B24] Defelipe L. A. Do Porto D. F. Pereira Ramos P. I. Nicolás M. F. Sosa E. Radusky L. (2016). A whole genome bioinformatic approach to determine potential latent phase specific targets in *Mycobacterium tuberculosis* . Mycobacterium Tuberc. Tuberc. Edinb. Scotl. 97, 181–192. 10.1016/j.tube.2015.11.009 26791267

[B25] Dewar C. E. MacGregor P. Cooper S. Gould M. K. Matthews K. R. Savill N. J. (2018). Mitochondrial DNA is critical for longevity and metabolism of transmission stage Trypanosoma brucei. PLoS Pathog. 14, e1007195. 10.1371/journal.ppat.1007195 30020996 PMC6066258

[B26] DiMasi J. A. Grabowski H. G. Hansen R. W. (2016). Innovation in the pharmaceutical industry: New estimates of R&D costs. J. Health Econ. 47, 20–33. 10.1016/j.jhealeco.2016.01.012 26928437

[B27] dos Santos P. F. Moreira D. S. Baba E. H. Volpe C. M. O. Ruiz J. C. Romanha A. J. (2016). Molecular characterization of lipoamide dehydrogenase gene in *Trypanosoma cruzi* populations susceptible and resistant to benznidazole. Exp. Parasitol. 170, 1–9. 10.1016/j.exppara.2016.08.006 27567984

[B28] Downey N. Hines J. C. Sinha K. M. Ray D. S. (2005). Mitochondrial DNA ligases of *Trypanosoma brucei* . Eukaryot. Cell 4, 765–774. 10.1128/EC.4.4.765-774.2005 15821136 PMC1087824

[B29] Dufernez F. Yernaux C. Gerbod D. Noël C. Chauvenet M. Wintjens R. (2006). The presence of four iron-containing superoxide dismutase isozymes in Trypanosomatidae: Characterization, subcellular localization, and phylogenetic origin in *Trypanosoma brucei* . Free Radic. Biol. Med. 40, 210–225. 10.1016/j.freeradbiomed.2005.06.021 16413404

[B30] Eastman R. T. Buckner F. S. Yokoyama K. Gelb M. H. Van Voorhis W. C. (2006). Thematic review series: Lipid Posttranslational Modifications. Fighting parasitic disease by blocking protein farnesylation. J. Lipid Res. 47, 233–240. 10.1194/jlr.R500016-JLR200 16339110

[B31] Ebersoll S. Musunda B. Schmenger T. Dirdjaja N. Bonilla M. Manta B. (2018). A glutaredoxin in the mitochondrial intermembrane space has stage-specific functions in the thermo-tolerance and proliferation of African trypanosomes. Redox Biol. 15, 532–547. 10.1016/j.redox.2018.01.011 29413965 PMC5975080

[B32] El-Sayed N. M. Myler P. J. Blandin G. Berriman M. Crabtree J. Aggarwal G. (2005). Comparative genomics of trypanosomatid parasitic Protozoa. Science 309, 404–409. 10.1126/science.1112181 16020724

[B33] Engstler M. Weise F. Bopp K. Grünfelder C. G. Günzel M. Heddergott N. (2005). The membrane-bound histidine acid phosphatase TbMBAP1 is essential for endocytosis and membrane recycling in *Trypanosoma brucei* . J. Cell Sci. 118, 2105–2118. 10.1242/jcs.02327 15855239

[B34] Farfán-López M. Espinoza-Culupú A. García-de-la-Guarda R. Serral F. Sosa E. Palomino M. M. (2020). Prioritisation of potential drug targets against Bartonella bacilliformis by an integrative *in-silico* approach. Mem. Inst. Oswaldo Cruz 115, e200184. 10.1590/0074-02760200184 32785422 PMC7416641

[B35] Faria J. Loureiro I. Santarém N. Macedo-Ribeiro S. Tavares J. Cordeiro-da-Silva A. (2016). *Leishmania infantum* asparagine synthetase A is dispensable for parasites survival and infectivity. PLoS Negl. Trop. Dis. 10, e0004365. 10.1371/journal.pntd.0004365 26771178 PMC4714757

[B36] Fenno J. C. Lee S. Y. Bayer C. H. Ning Y. (2001). The opdB locus encodes the trypsin-like peptidase activity of Treponema denticola. Infect. Immun. 69, 6193–6200. 10.1128/IAI.69.10.6193-6200.2001 11553560 PMC98751

[B37] Ferri G. Edreira M. M. (2021). All roads lead to cytosol: Trypanosoma cruzi multi-strategic approach to invasion. Front. Cell. Infect. Microbiol. 11, 634793. 10.3389/fcimb.2021.634793 33747982 PMC7973469

[B38] Ghosh S. Goswami S. Adhya S. (2003). Role of superoxide dismutase in survival of Leishmania within the macrophage. Biochem. J. 369, 447–452. 10.1042/BJ20021684 12459037 PMC1223130

[B39] Girard R. M. B. M. Crispim M. Stolić I. Damasceno F. S. Santos da Silva M. Pral E. M. F. (2016). An aromatic diamidine that targets kinetoplast DNA, impairs the cell cycle in trypanosoma cruzi, and diminishes trypomastigote release from infected mammalian host cells. Antimicrob. Agents Chemother. 60, 5867–5877. 10.1128/AAC.01595-15 27431229 PMC5038236

[B40] Guo Z.-J. Lamb C. Dixon R. A. (1998). A serine protease from suspension-cultured soybean cells. Phytochemistry 47, 547–553. 10.1016/S0031-9422(97)00441-X 9461673

[B41] Hotez P. J. Molyneux D. H. Fenwick A. Kumaresan J. Sachs S. E. Sachs J. D. (2007). Control of neglected tropical diseases. N. Engl. J. Med. 357, 1018–1027. 10.1056/NEJMra064142 17804846

[B42] Inbar E. Hughitt V. K. Dillon L. A. L. Ghosh K. El-Sayed N. M. Sacks D. L. (2017). The transcriptome of Leishmania major developmental stages in their natural sand fly vector. mBio 8. 10.1128/mBio.00029-17 PMC5380837 28377524

[B43] Jensen R. E. Englund P. T. (2012). Network news: The replication of kinetoplast DNA. Annu. Rev. Microbiol. 66, 473–491. 10.1146/annurev-micro-092611-150057 22994497

[B44] Jones P. M. George A. M. (2004). The ABC transporter structure and mechanism: Perspectives on recent research. Cell. Mol. Life Sci. CMLS 61, 682–699. 10.1007/s00018-003-3336-9 15052411 PMC11138499

[B45] Kagan V. E. Shvedova A. Serbinova E. Khan S. Swanson C. Powell R. (1992). Dihydrolipoic acid-a universal antioxidant both in the membrane and in the aqueous phase. Reduction of peroxyl, ascorbyl and chromanoxyl radicals. Biochem. Pharmacol. 44, 1637–1649. 10.1016/0006-2952(92)90482-X 1417985

[B46] Karp P. D. Paley S. Romero P. (2002). The pathway tools software. Bioinforma. Oxf. Engl. 18 (1), S225–S232. 10.1093/bioinformatics/18.suppl_1.s225 12169551

[B47] Krauth‐Siegel R. L. Schöneck R. (1995). Flavoprotein structure and mechanism. 5. Trypanothione reductase and lipoamide dehydrogenase as targets for a structure-based drug design. FASEB J. 9, 1138–1146. 10.1096/fasebj.9.12.7672506 7672506

[B48] Law J. A. O’Hearn S. Sollner-Webb B. (2007). In Trypanosoma brucei RNA editing, TbMP18 (band VII) is critical for editosome integrity and for both insertional and deletional cleavages. Mol. Cell. Biol. 27, 777–787. 10.1128/MCB.01460-06 17101787 PMC1800803

[B49] Li Y. Shah-Simpson S. Okrah K. Belew A. T. Choi J. Caradonna K. L. (2016). Transcriptome remodeling in *Trypanosoma cruzi* and human cells during intracellular infection. PLOS Pathog. 12, e1005511. 10.1371/journal.ppat.1005511 27046031 PMC4821583

[B50] Lohrer H. Krauth-Siegel R. L. (1990). Purification and characterization of lipoamide dehydrogenase from *Trypanosoma cruzi* . Eur. J. Biochem. 194, 863–869. 10.1111/j.1432-1033.1990.tb19480.x 2269305

[B51] Loureiro I. Faria J. Clayton C. Ribeiro S. M. Roy N. Santarém N. (2013). Knockdown of asparagine synthetase A renders *Trypanosoma brucei* auxotrophic to asparagine. PLoS Negl. Trop. Dis. 7, e2578. 10.1371/journal.pntd.0002578 24340117 PMC3854871

[B52] Lukeš J. Skalický T. Týč J. Votýpka J. Yurchenko V. (2014). Evolution of parasitism in kinetoplastid flagellates. Mol. Biochem. Parasitol. 195, 115–122. 10.1016/j.molbiopara.2014.05.007 24893339

[B53] Madison-Antenucci S. Grams J. Hajduk S. L. (2002). Editing machines: The complexities of trypanosome RNA editing. Cell 108, 435–438. 10.1016/s0092-8674(02)00653-0 11909515

[B54] Manhas R. Tripathi P. Khan S. Sethu Lakshmi B. Lal S. K. Gowri V. S. (2014). Identification and functional characterization of a novel bacterial type asparagine synthetase A: A tRNA synthetase paralog from Leishmania donovani. J. Biol. Chem. 289, 12096–12108. 10.1074/jbc.M114.554642 24610810 PMC4002115

[B55] Manzano J. I. Perea A. León-Guerrero D. Campos-Salinas J. Piacenza L. Castanys S. (2017). Leishmania LABCG1 and LABCG2 transporters are involved in virulence and oxidative stress: Functional linkage with autophagy. Parasit. Vectors 10, 267. 10.1186/s13071-017-2198-1 28558770 PMC5450059

[B56] Marquez V. E. Arias D. G. Piattoni C. V. Robello C. Iglesias A. A. Guerrero S. A. (2010). Cloning, expression, and characterization of a dithiol glutaredoxin from *Trypanosoma cruzi* . Antioxid. Redox Signal. 12, 787–792. 10.1089/ars.2009.2907 19769456

[B57] Martínez A. Prolo C. Estrada D. Rios N. Alvarez M. N. Piñeyro M. D. (2019). Cytosolic Fe-superoxide dismutase safeguards *Trypanosoma cruzi* from macrophage-derived superoxide radical. Proc. Natl. Acad. Sci. U. S. A. 116, 8879–8888. 10.1073/pnas.1821487116 30979807 PMC6500117

[B58] Matheson N. Schmidt J. Travis J. (1995). Isolation and properties of an angiotensin II-cleaving peptidase from mesquite pollen. Am. J. Respir. Cell Mol. Biol. 12, 441–448. 10.1165/ajrcmb.12.4.7695924 7695924

[B59] Matthews K. R. (2005). The developmental cell biology of *Trypanosoma brucei* . J. Cell Sci. 118, 283–290. 10.1242/jcs.01649 15654017 PMC2686837

[B60] McCord J. M. Fridovich I. (1969). Superoxide dismutase. J. Biol. Chem. 244, 6049–6055. 10.1016/S0021-9258(18)63504-5 5389100

[B61] Mesu V. K. B. K. Kalonji W. M. Bardonneau C. Mordt O. V. Blesson S. Simon F. (2018). Oral fexinidazole for late-stage african *Trypanosoma brucei gambiense* trypanosomiasis: A pivotal multicentre, randomised, non-inferiority trial. Lancet 391, 144–154. 10.1016/S0140-6736(17)32758-7 29113731

[B62] Mittra B. Laranjeira-Silva M. F. Miguel D. C. Perrone Bezerra de Menezes J. Andrews N. W. (2017). The iron-dependent mitochondrial superoxide dismutase SODA promotes Leishmania virulence. J. Biol. Chem. 292, 12324–12338. 10.1074/jbc.M116.772624 28550086 PMC5519379

[B63] Morty R. E. Fülöp V. Andrews N. W. (2002). Substrate recognition properties of oligopeptidase B from *Salmonella enterica serovar typhimurium* . J. Bacteriol. 184, 3329–3337. 10.1128/JB.184.12.3329-3337.2002 12029050 PMC135088

[B64] Morty R. E. Lonsdale-Eccles J. D. Mentele R. Auerswald E. A. Coetzer T. H. T. (2001). Trypanosome-Derived oligopeptidase B is released into the plasma of infected rodents, where it persists and retains full catalytic activity. Infect. Immun. 69, 2757–2761. 10.1128/IAI.69.4.2757-2761.2001 11254649 PMC98221

[B65] Morty R. E. Shih A. Y. Fülöp V. Andrews N. W. (2005). Identification of the reactive cysteine residues in oligopeptidase B from *Trypanosoma brucei* . FEBS Lett. 579, 2191–2196. 10.1016/j.febslet.2005.03.014 15811340

[B66] Motta M. C. M. (2008). Kinetoplast as a potential chemotherapeutic target of trypanosomatids. Curr. Pharm. Des. 14, 847–854. 10.2174/138161208784041051 18473834

[B67] Naguleswaran A. Doiron N. Roditi I. (2018). RNA-Seq analysis validates the use of culture-derived Trypanosoma brucei and provides new markers for mammalian and insect life-cycle stages. BMC Genomics 19, 227. 10.1186/s12864-018-4600-6 29606092 PMC5879877

[B68] Nogueira F. B. Krieger M. A. Nirdé P. Goldenberg S. Romanha A. J. Murta S. M. F. (2006). Increased expression of iron-containing superoxide dismutase-A (TcFeSOD-A) enzyme in *Trypanosoma cruzi* population with *in vitro*-induced resistance to benznidazole. Acta Trop. 100, 119–132. 10.1016/j.actatropica.2006.10.004 17113553

[B69] Nozaki T. Engel J. C. Dvorak J. A. (1996). Cellular and molecular biological analyses of nifurtimox resistance in *Trypanosoma cruzi* . Am. J. Trop. Med. Hyg. 55, 111–117. 10.4269/ajtmh.1996.55.111 8702014

[B70] Ohkanda J. Buckner F. S. Lockman J. W. Yokoyama K. Carrico D. Eastman R. (2004). Design and synthesis of peptidomimetic protein farnesyltransferase inhibitors as anti- *Trypanosoma brucei* agents. J. Med. Chem. 47, 432–445. 10.1021/jm030236o 14711313

[B71] Oliveira L. F. Schubach A. O. Martins M. M. Passos S. L. Oliveira R. V. Marzochi M. C. (2011). Systematic review of the adverse effects of cutaneous leishmaniasis treatment in the New World. Acta Trop. 118, 87–96. 10.1016/j.actatropica.2011.02.007 21420925

[B72] Palumbo M. Sosa E. Castello F. Schottlender G. Serral F. Turjanski A. (2022). Integrating diverse layers of omic data to identify novel drug targets in Listeria monocytogenes. Front. Drug Discov. 2. 10.3389/fddsv.2022.969415

[B73] Pérez-Molina J. A. Molina I. (2018). Chagas disease. Lancet 391, 82–94. 10.1016/S0140-6736(17)31612-4 28673423

[B74] Polgár L. (1997). A potential processing enzyme in prokaryotes: Oligopeptidase B, a new type of serine peptidase. Proteins Struct. Funct. Bioinforma. 28, 375–379. 10.1002/(SICI)1097-0134(199707)28:3<375::AID-PROT7>3.0.CO;2-B 9223183

[B75] Portela M. P. Stopopani A. O. (1991). Lipoamide dehydrogenase from *Trypanosoma cruzi*: Some properties and cellular localization. Biochem. Int. 24, 147–155. 1768255

[B76] Qi Y. Grishin N. V. (2004). Structural classification of thioredoxin-like fold proteins. Proteins Struct. Funct. Bioinforma. 58, 376–388. 10.1002/prot.20329 15558583

[B77] Rajan K. S. Doniger T. Cohen-Chalamish S. Chen D. Semo O. Aryal S. (2019). Pseudouridines on *Trypanosoma brucei* spliceosomal small nuclear RNAs and their implication for RNA and protein interactions. Nucleic Acids Res. 47, 7633–7647. 10.1093/nar/gkz477 31147702 PMC6698659

[B78] Ramos P. I. P. Fernández Do Porto D. Lanzarotti E. Sosa E. J. Burguener G. Pardo A. M. (2018). An integrative, multi-omics approach towards the prioritization of *Klebsiella pneumoniae* drug targets. Sci. Rep. 8, 10755. 10.1038/s41598-018-28916-7 30018343 PMC6050338

[B79] Rodriques Coura J. de Castro S. L. (2002). A critical review on Chagas disease chemotherapy. Mem. Inst. Oswaldo Cruz 97, 3–24. 10.1590/s0074-02762002000100001 11992141

[B80] Roldán A. Comini M. A. Crispo M. Krauth-Siegel R. L. (2011). Lipoamide dehydrogenase is essential for both bloodstream and procyclic *Trypanosoma brucei: Trypanosoma brucei* lipoamide dehydrogenase. Mol. Microbiol. 81, 623–639. 10.1111/j.1365-2958.2011.07721.x 21631607

[B81] Salavati R. Moshiri H. Kala S. Shateri Najafabadi H. (2011). Inhibitors of RNA editing as potential chemotherapeutics against trypanosomatid pathogens. Int. J. Parasitol. Drugs Drug Resist. 2, 36–46. 10.1016/j.ijpddr.2011.10.003 24533263 PMC3862403

[B82] Sanz A. M. Gómez-Contreras F. Navarro P. Sánchez-Moreno M. Boutaleb-Charki S. Campuzano J. (2008). Efficient inhibition of iron superoxide dismutase and of trypanosoma cruzi growth by benzo[ g ]phthalazine derivatives functionalized with one or two imidazole rings. J. Med. Chem. 51, 1962–1966. 10.1021/jm701179m 18293910

[B83] Schmidtke P. Barril X. (2010). Understanding and predicting druggability. A high-throughput method for detection of drug binding sites. J. Med. Chem. 53, 5858–5867. 10.1021/jm100574m 20684613

[B84] Schmidtke P. Le Guilloux V. Maupetit J. Tuffery P. (2010). fpocket: online tools for protein ensemble pocket detection and tracking. Nucleic Acids Res. 38, W582–W589. 10.1093/nar/gkq383 20478829 PMC2896101

[B85] Schnaufer A. Domingo G. J. Stuart K. (2002). Natural and induced dyskinetoplastic trypanosomatids: How to live without mitochondrial DNA. Int. J. Parasitol. 32, 1071–1084. 10.1016/s0020-7519(02)00020-6 12117490

[B86] Schöneck R. Billaut-Mulot O. Numrich P. Ouaissi M. A. Krauth-Siegel R. L. (1997). Cloning, sequencing and functional expression of dihydrolipoamide dehydrogenase from the human pathogen *Trypanosoma cruzi* . Eur. J. Biochem. 243, 739–747. 10.1111/j.1432-1033.1997.00739.x 9057840

[B87] Schottlender G. Prieto J. M. Palumbo M. C. Castello F. A. Serral F. Sosa E. J. (2022). From drugs to targets: Reverse engineering the virtual screening process on a proteomic scale. Front. Drug Discov. 2. 10.3389/fddsv.2022.969983

[B88] Serral F. Castello F. A. Sosa E. J. Pardo A. M. Palumbo M. C. Modenutti C. (2021). From genome to drugs: New approaches in antimicrobial discovery. Front. Pharmacol. 12, 647060. 10.3389/fphar.2021.647060 34177572 PMC8219968

[B89] Serral F. Pardo A. M. Sosa E. Palomino M. M. Nicolás M. F. Turjanski A. G. (2022). Pathway driven target selection in *Klebsiella pneumoniae*: Insights into carbapenem exposure. Front. Cell. Infect. Microbiol. 12, 773405. 10.3389/fcimb.2022.773405 35174104 PMC8841789

[B90] Sosa E. J. Burguener G. Lanzarotti E. Defelipe L. Radusky L. Pardo A. M. (2018). Target-pathogen: A structural bioinformatic approach to prioritize drug targets in pathogens. Nucleic Acids Res. 46, D413–D418. 10.1093/nar/gkx1015 29106651 PMC5753371

[B91] Souza W. (2002). Basic cell biology of *Trypanosoma cruzi* . Curr. Pharm. Des. 8, 269–285. 10.2174/1381612023396276 11860366

[B92] Steinegger M. Söding J. (2017). MMseqs2 enables sensitive protein sequence searching for the analysis of massive data sets. Nat. Biotechnol. 35, 1026–1028. 10.1038/nbt.3988 29035372

[B93] Stuart K. D. Schnaufer A. Ernst N. L. Panigrahi A. K. (2005). Complex management: RNA editing in trypanosomes. Trends biochem. Sci. 30, 97–105. 10.1016/j.tibs.2004.12.006 15691655

[B94] Sundar S. Chakravarty J. (2010). Antimony toxicity. Int. J. Environ. Res. Public. Health 7, 4267–4277. 10.3390/ijerph7124267 21318007 PMC3037053

[B95] Swenerton R. K. Zhang S. Sajid M. Medzihradszky K. F. Craik C. S. Kelly B. L. (2011). The oligopeptidase B of Leishmania regulates parasite enolase and immune evasion. J. Biol. Chem. 286, 429–440. 10.1074/jbc.M110.138313 20961853 PMC3013002

[B96] Tarun S. Z. Schnaufer A. Ernst N. L. Proff R. Deng J. Hol W. (2008). KREPA6 is an RNA-binding protein essential for editosome integrity and survival of *Trypanosoma brucei* . RNA 14, 347–358. 10.1261/rna.763308 18065716 PMC2212256

[B97] Teixeira D. E. Benchimol M. Rodrigues J. C. F. Crepaldi P. H. Pimenta P. F. P. de Souza W. (2013). The cell biology of Leishmania: How to teach using animations. PLoS Pathog. 9, e1003594. 10.1371/journal.ppat.1003594 24130476 PMC3795027

[B98] Wang J. Yao X. Huang J. (2017). New tricks for human farnesyltransferase inhibitor: Cancer and beyond. MedChemComm 8, 841–854. 10.1039/c7md00030h 30108801 PMC6072492

[B99] Wilkinson S. R. Taylor M. C. Horn D. Kelly J. M. Cheeseman I. (2008). A mechanism for cross-resistance to nifurtimox and benznidazole in trypanosomes. Proc. Natl. Acad. Sci. U. S. A. 105, 5022–5027. 10.1073/pnas.0711014105 18367671 PMC2278226

[B100] Willert E. K. Fitzpatrick R. Phillips M. A. (2007). Allosteric regulation of an essential trypanosome polyamine biosynthetic enzyme by a catalytically dead homolog. Proc. Natl. Acad. Sci. 104, 8275–8280. 10.1073/pnas.0701111104 17485680 PMC1895940

[B101] Willert E. K. Phillips M. A. (2009). Cross-species activation of trypanosome S-adenosylmethionine decarboxylase by the regulatory subunit prozyme. Mol. Biochem. Parasitol. 168, 1–6. 10.1016/j.molbiopara.2009.05.009 19523496 PMC2730992

[B102] Willert E. K. Phillips M. A. (2008). Regulated expression of an essential allosteric activator of polyamine biosynthesis in african trypanosomes. PLOS Pathog. 4, e1000183. 10.1371/journal.ppat.1000183 18949025 PMC2562514

[B103] Yan J.-B. Wang G.-Q. Du P. Zhu D.-X. Wang M.-W. Jiang X.-Y. (2006). High-level expression and purification of *Escherichia coli* oligopeptidase B. Purif 47, 645–650. 10.1016/j.pep.2006.01.018 16515865

[B104] Yang G. Choi G. No J. H. (2016). Antileishmanial mechanism of diamidines involves targeting kinetoplasts. Antimicrob. Agents Chemother. 60, 6828–6836. 10.1128/AAC.01129-16 27600039 PMC5075092

[B105] Yazaki E. Ishikawa S. A. Kume K. Kumagai A. Kamaishi T. Tanifuji G. (2017). Global Kinetoplastea phylogeny inferred from a large-scale multigene alignment including parasitic species for better understanding transitions from a free-living to a parasitic lifestyle. Genes Genet. Syst. 92, 35–42. 10.1266/ggs.16-00056 28216511

[B106] Yeh I. Hanekamp T. Tsoka S. Karp P. D. Altman R. B. (2004). Computational analysis of plasmodium falciparum metabolism: Organizing genomic information to facilitate drug discovery. Genome Res. 14, 917–924. 10.1101/gr.2050304 15078855 PMC479120

[B107] Yokoyama K. Trobridge P. Buckner F. S. Scholten J. Stuart K. D. Van Voorhis W. C. (1998). The effects of protein farnesyltransferase inhibitors on trypanosomatids: Inhibition of protein farnesylation and cell growth. Mol. Biochem. Parasitol. 94, 87–97. 10.1016/S0166-6851(98)00053-X 9719512

[B108] Zhang F. L. Casey P. J. (1996). Protein prenylation: Molecular mechanisms and functional consequences. Annu. Rev. Biochem. 65, 241–269. 10.1146/annurev.bi.65.070196.001325 8811180

[B109] Zingales B. Araujo R. G. A. Moreno M. Franco J. Aguiar P. H. N. Nunes S. L. (2015). A novel ABCG-like transporter of *Trypanosoma cruzi* is involved in natural resistance to benznidazole. Mem. Inst. Oswaldo Cruz 110, 433–444. 10.1590/0074-02760140407 25946152 PMC4489481

